# Gamma knife radiosurgery of meningiomas involving the foramen magnum

**DOI:** 10.4103/0974-8237.65478

**Published:** 2010

**Authors:** Robert M. Starke, James H. Nguyen, Davis L. Reames, Jessica Rainey, Jason P. Sheehan

**Affiliations:** Department of Neurological Surgery, University of Virginia, Charlottesville, VA, USA

**Keywords:** Foramen magnum, gamma knife, meningioma, microsurgery, outcome, radiosurgery, recurrence, skull base

## Abstract

**Background:**

Foramen magnum meningiomas represent a challenging clinical entity. Although resection is performed for those with a mass effect, complete resection is not always feasible. For some patients, stereotactic radiosurgery may be used as the primary treatment modality. We evaluatedthe long-term outcome of Gamma Knife radiosurgery (GKRS) for the treatment of patientswith a foramen magnum meningioma.

**Materials and Methods:**

Between 1991 and 2005, 222 patients with a meningioma in the posterior fossa were treated with GKRS at the University of Virginia. Of these patients, 5 had meningiomas involving the foramen magnum. At the time of GKRS, the median age of the patients was 60 years (range, 51–78). Three patients were treated with radiosurgery following an initial resection and 2 were treated with upfront radiosurgery. The patients were assessed clinically and radiologically at routine intervals following GKRS.

**Results:**

The median tumor volume was 6.8 cc (range 1.9–17 cc). The GKRS tumor received a marginal dose of 12 Gy (range 10–15), and the median number of isocenters was 5 (range 3–19). The mean follow-up was 6 years (range 4–13). One lesion increased in size following GKRS requiring a second treatment, resulting in size stabilization. At the time of the last follow-up, all meningiomas had either demonstrated no growth (n = 4) or reduction in size (n = 1). No patients experienced post-radiotherapy complications.

**Conclusions:**

GKRS affords a high rate of tumor control and preservation of neurologic function for patients with foramen magnum meningiomas. Further study of its role in the neurosurgical management of such patients seems warranted.

## INTRODUCTION

The foramen magnum is the site of tumor origin in 1.8–3.2% of intracranial meningiomas and constitutes approximately 6.5% of posterior cranial fossa meningiomas.[1,2] The first successful resection of a foramen magnum meningioma was accomplished by Elsberg and Strauss in 1927 via a suboccipital craniotomy and C1–C3 laminectomy.[3] Since then, many surgical approaches have been applied. These tumors require careful surgical manipulation as they are often in close proximity to critical vascular and neural structures. Reported complete resection rates of these tumors vary significantly in the literature and are often associated with significant morbidity, recurrence, and mortality.[1,2,4‐27]

Although there have been many studies describing the microsurgical resection of foramen magnum meningiomas, reports regarding the complications and long-term outcomes of patients treated with primary or adjuvant radiosurgery are sparse. In a select group of patients with small lesions in which imaging, clinical history, and the patient examination are consistent with a meningioma, radiation may be used as a primary treatment option or as an adjuvant treatment following an incomplete resection. Primary radiosurgery may be an alternative treatment in patients with advanced age or medical comorbidity, high operative risks, or in those who refuse surgery or have residual or recurrent tumors. In this article, we present the long-term outcomes of patients with meningiomas involving the foramen magnum, treated with both primary and adjuvant radiosurgery.

## MATERIALS AND METHODS

Between 1991 and 2005, 222 patients underwent Gamma Knife radiosurgery (GKRS) for posterior fossa meningiomas at the University of Virginia. Of these patients, 5 had meningioma involving the foramen magnum and a minimum follow-up of 2 years. The diagnosis was confirmed either by tissue pathology or characteristic findings on neuroimaging. An Institutional Review Board–approved review of these patients was undertaken in order to collect data on various patient and tumor characteristics, including age, gender, presenting symptoms, tumor volume, and radiosurgical parameters.

Patients were considered for radiosurgery if the lesion was less than 35 mm in maximal dimension and if the patient was not disabled by the tumor. Patients were not candidates for primary surgical treatment based on their advanced age, the projected operative risks based on medical comorbidities, and/or refusal of microsurgical resection. Adjuvant radiosurgery was carried out in patients with regrowth of lesions following microsurgical excision or as part of multimodality treatment whereby the risks of complete surgical resection outweighed the benefits of multimodality therapy.

### Patient attributes

Five women received GKRS of a meningioma involving the foramen magnum. The median age of the patients was 60 years (range, 51–78 years). The median duration from the initial onset of symptoms to GKRS was 60 months (range 48–126 months). The most common symptoms on presentation were headache (n = 3), dizziness/imbalance (n = 3), body hyperesthesia/hypoesthesia/paresthesia (n = 3), dysphagia (n = 2), and hemiparesis/weakness (n = 2). The median patient Karnofsky Performance Score prior to treatment was 75% (range 60–90%).

### Lesion characteristics

Four lesions extended along the clivus or brainstem into the anterior foramen magnum, whereas a single lesion extended along the posterior foramen magnum. On imaging before GKS therapy, the median volume of lesions was 6.8 cc (mean 7.4, range 1.9–17). No lesions had significant associated edema.

### Radiosurgical planning

All the patients were treated using the Gamma Knife Model U unit or the Model C unit (Elekta Instruments, Stockholm, Sweden). The Model C unit replaced the model U unit in 2001. Stereotactic frame placement took place in the operating room under local anesthesia. A contrast-enhanced, 1.5 T, T1-weighted magnetic resonance (MR) image was obtained for treatment planning using 1–1.5 mm axial slice thickness with no gap separation. In 1 patient, a contrast-enhanced, 1-mm axial slice thickness computed tomography (CT) image was obtained because MR imaging was contraindicated. GammaPlan software (Elekta Instruments) was used for dose planning in all the cases.

The treatment regimen was determined by the attending neurosurgeon, radiation oncologist, and medical physicist. The treatment isodose, central tumor dose, and tumor margin dose were guided by the evaluation of the size, location, and the projected risk to the adjacent structures. The median maximum tumor dose was 30 Gy (mean, 28.6 Gy; range, 20–40 Gy). The median marginal dose was 12 Gy (mean, 11.8; range, 10–15 Gy). Multiple isocenters of irradiation were required for all the patients to appropriately cover the irregular architecture of each tumor (median, 5; mean, 8; range, 3–19). The treatment regimens used for these lesions are given in Table 1.

**Table 1:** Gamma knife radiosurgery parameters

Characteristics	Median (mean, range)
Number of isodose centers	5 (7.8, 3–19)
Margin dose (Gy)	12 (11.8, 10–15)
Maximum dose (Gy)	30 (28.6, 20–40)
Isodose (%)	40 (40, 30–75
Prescription dose (mL)	6.2 (7.1, 1.9–12.4)
Conformity index^1^	1.3 (1.1, 0.7–1.7)

1 Prescription dose/tumor volume

### Patient follow-up

Follow-up imaging was requested at 6-month intervals for the first 2 years and yearly thereafter. Follow-up ranged from 4 to 13 years (mean, 6; median, 5). A decrease or increase in tumor size was defined as a 15% or greater change in tumor volume. The clinical follow-up consisted of chart review and/or physician office follow-up letters.

### Case descriptions

#### Case 1

A 60-year-old female with a long history of severe headaches and intermittent nausea and vomiting presented with weakness of the left lower extremity, numbness in the right upper extremity, and difficulty with ambulation. A CT scan revealed a large foramen magnum tumor. The patient underwent a suboccipital craniectomy with a subtotal resection of the tumor. A 6-month postoperative MRI revealed a partially calcified meningioma arising from the dorsal clivus extending down through the anterior foramen magnum to the level of the first cervical vertebra. At this time, the patient was experiencing dysphagia, diplopia, right hemiparesis, left hemisensory loss, and severe imbalance.

At the time of GKRS 8 months after surgery, the volume of the tumor was approximately 7.5 cc. Five isocenters were used for targeting the tumor, and the tumor received a maximum dose of 20 Gy with the brainstem receiving approximately 10–20% of this dose. At 51-month follow-up after GKRS, she noted improvement in her symptoms and imaging revealed no growth of the meningioma.

#### Case 2

A 59-year-old female presented with dizziness, imbalance, ataxia, and hypoesthesia of the face and body bilaterally. Imaging was consistent with a meningioma extending en plaque from the junction of the medulla and the pons into the foramen magnum along the clivus. The patient underwent surgery for partial resection of the tumor, and the residual tumor was treated with GKRS.

**Figure 1:**
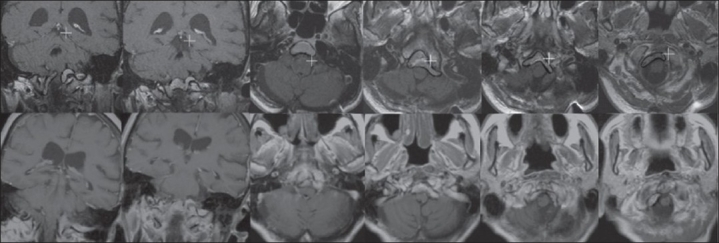
Row 1: At the time of the gamma knife radiosurgery (GKRS), the patient had a meningioma with a volume of 6.8 cc extending en plaque from the junction of the medulla and the pons into the foramen magnum along the clivus. Row 2: On follow-up imaging 13 years after GKRS, the lesion decreased in size to 4.0 cc without any new growth or radiographic complications of GKRS (cyst formation, necrosis, and edema)

MRI at the time of GKRS demonstrated a tumor measuring 6.8 cc in volume [Figure 1]. Eight isocenters were targeted, and the tumor received a margin dose of 10 Gy at the 50% isodose line and a maximum dose of 20 Gy. A follow-up MRI 13 years after the GKRS revealed that the tumor had decreased in volume to 4 cc, demonstrating a 41% decrease in comparison with the initial treatment image. In the last few years, the patient had some increase in her imbalance and dizziness, but without radiographic evidence of edema, cyst formation, or necrosis. The patient recently died due to unrelated comorbidities.

#### Case 3

A 51-year-old female had an incidental finding of meningioma while being evaluated for pain and headaches after a motor vehicle accident. Imaging revealed a meningioma in the occipital region contiguous with the torcula superiorly and extending into the foramen magnum inferiorly. The patient elected to undergo GKRS therapy. At the time of treatment, she was asymptomatic except for occasional headaches and paresthesia of the proximal left arm.

At the time of treatment, the tumor measured 1.9 cc in volume. Four isocenters were used to deliver a conformal radiosurgical treatment, and the tumor received a marginal dose of 12 Gy at the 30% isodose line and a maximum dose of 40 Gy. The tumor's volume measured 1.8 cc on a follow-up MR image 48 months after the treatment, and the patient had no residual symptoms.

#### Case 4

A 61-year-old female was found to have a left hemispheric meningioma extending from the parasellar region to the foramen magnum during a work-up for chronic headaches. She underwent a subtotal resection of the tumor and was referred to our institution for GKRS. At the time of her presentation to our center for treatment of her residual lesion 3 months later, she was experiencing occasional headache, facial hypoesthesia, trigeminal neuralgia, impaired abduction of her left eye, and decreased hearing in her left ear.

The volume of the tumor was 17 cc at the time of her GKRS. Nineteen isocenters were targeted, and 30 Gy were given as a maximum dose. The tumor received a margin dose of 15 Gy at the 50% isodose line. Follow-up MR imaging 73 months later revealed a tumor volume of 17 cc, indicating no change in tumor size after radiosurgery. During clinical follow-up 82 months after GKRS treatment, she found improvement in her headaches. She continued to have occasional episodes of trigeminal neuralgia which decreased with medical therapy. She also had a number of seizures in the first 4 years after GKRS, but without radiographic evidence of edema, cyst formation, or necrosis related to GKRS. The patient was started on medical therapy and had not had a seizure in the last 6 years.

#### Case 5

A 74-year-old female with 3 previous resections for a pituitary adenoma was found to have a cervicomedullary junction meningioma. The patient was referred to our center with a preference to undergo GKRS. On presentation, she had slurred speech, vertigo, and hypoesthesia and weakness in her right hand.

The tumor measured 3.9 cc at the time of her GKS treatment and extending down through the foramen magnum to the second cervical vertebral level. Three isocenters were used to target the lesion; 24 Gy was given as a maximum dose, and a marginal dose of 15 Gy was given to the 30% isodose line. The vascular supply of the tumor received a dose of 24 Gy.

Follow-up imaging 48 months after the treatment revealed that the tumor had grown in size to 5.5 cc, indicating a 41% increase. Her symptoms remained unchanged. At this time, a second GKRS treatment was carried out. Three isocenters were again used to target the tumor, and the lesion received a maximum dose of 33.33 Gy and a marginal dose of 17 Gy to the 30% isodose line. Follow-up imaging 12 months later revealed a tumor volume of 5.5 cc, indicating no change in the size of the tumor since her second treatment.

## DISCUSSION

Meningiomas are the most common lesions located in the foramen magnum region, and they account for 60–75% of tumors in this location.[2,28,29] Most lesions are in the anterior or anterolateral intradural space, but they may arise posterolaterally as well. An en plaque variety with extradural extension also exists.[7] These are slow growing lesions that commonly cause significant distortion of the brainstem or spinal cord and often encase key vascular structures and cranial nerves by the time of presentation. For this reason, the majority of patients have multiple symptoms and/or deficits on presentation. The classic foramen magnum syndrome describes motor and sensory deficits developing first in one arm with progression to the ipsilateral leg prior to involvement of the contralateral leg before completing the progression in the contralateral arm. Although this clockwise symptomatology is well associated with these lesions, common presentations simply involve occipital headaches and neck pain with a predominance of cerebellar and spinal cord dysfunction.[1,2,4‐26] In our series, headache or headache and cervical pain, dizziness or imbalance, and alterations in sensation were the most common presenting symptoms.

As a result of the complex nature and difficult location of these lesions, many surgical approaches have been used to resect these lesions, including anterior–lateral transcervical, transoral, posterolateral (retrocondylar), and far lateral (transcondylar).[1,2,4‐26] The first studies concerning surgical treatment of foramen magnum meningiomas often showed disappointing results with mortality rates of 19%[30] and 45%.[31] With the advent of microsurgery, mortality rates decreased, but mortality rates of 10–30% were not uncommon.[9,19,32] With refinements in surgical techniques, patient selection, more conservative operative plans and approaches, and adjuvant radiosurgery, surgical series have reported surgical mortality rates of 0–10%.[2,4,7,11,12,18,25,27,28,32] Nevertheless, these patients often experience a significant morbidity due to cranial nerve injury and damage to critical vasculature, brain stem, and spinal cord. The resulting deficits can be severe and complications may include hypertension, respiratory depression, aspiration, pneumonia, and mediastinitis among others.[1,2,4‐26]

Similarly, reported complete resection rates of tumors of foramen magnum (Simpson grade 1–2) range from 40% to 96% and are dependent on many characteristics of the tumor. In one series, intradural-based lesions could be resected in 94% of the cases, whereas tumors with extradural components could be resected in only 50% of the cases. Recurrence rates range from 12% to 91% and depend on a number of variables, including tumor characteristics, treatment paradigms, and follow-up.[1,2,4‐26]

The high recurrence rates of lesions in this location foretell a less than favorable prognosis. In the series by Stein *et al*., 20% of the patients died because of problems related to tumor recurrences.[33] In the series by Meyer *et al*., 5% of the patients died subsequent to tumor recurrence within 3 years of surgery.[27] In the majority of reports, re-operations for recurrent tumors could only be subtotal and were related to a significant postoperative neurologic compromise.[2,6,7,16,17,22,25] Tumors with adherence to the brainstem, encasement of the vertebral arteries and/or cranial nerves, en plaque growth pattern, high tumor grade, high mitotic activity/Ki-67 labeling, and loss of 1p36.1–p34 have been associated with an increased rate of incomplete resection and/or tumor recurrence.[34,35]

In a significant number of cases, complete surgical resection is not possible,[34,36] and the best follow-up treatment remains unclear. Previous authors have recommended radical resection in recurrent meningiomas of the foramen magnum to prolong life in neurologically stable patients.[6] Others argue that radiosurgery may be used as an adjunctive treatment after subtotal tumor resection or to arrest the progression of recurrent tumors.[14,37‐39] In these complex lesions, the treatment plan is often to achieve the most aggressive surgical resection with the goal of preserving full function using radiosurgery for residual tumors.[14,37‐39]

Although there have been more than 40 published articles concerning the operative outcomes of meningiomas of the foramen magnum, there is a paucity of literature concerning radiosurgery of lesions extending into the foramen magnum. Additionally, the majority of series note the use of radiosurgery of these lesions without having evidence or sources to define the outcomes and complications of radiosurgery of meningiomas of the foramen magnum.

Nicolato *et al*. reported the use of radiosurgery in the treatment of 62 meningiomas of the posterior fossa.[40] Only 1 lesion involved the foramen magnum. During a follow-up of 6–64.3 months (median, 28.7 months), neuroimaging evaluation documented the disappearance or reduction of the meningioma mass in 34/62 (55%) cases, a stable imaging in 25/62 (40%), and progression in 3/62 (5%). Two patients died from tumor progression, and 6.5% of the patients experienced transient complications due to post-radiosurgical edema.

We believe that radiosurgery is an alternative treatment in patients with advanced age, high operative risks, and in those who refuse surgery or have residual or recurrent tumors. In a select group of patients with small lesions in which imaging is diagnostic of meningiomas, radiation may be used as a primary treatment option. In our series of 5 patients, 4 had a decrease or no increase in the size of their lesions (follow-up range, 4–13 years). The fifth patient achieved had imaging confirming tumor progression. The patient achieved stabilization aftera second treatment with GKRS. One patient experienced intermittent seizures during the following microsurgery and GKRS that were medically controlled and was without seizures in the last 6 years of follow-up. No other patient experienced symptoms or complications related to radiosurgery.

To our knowledge, there is only one other similar report of foramen magnum meningiomas treated with radiosurgery.[41] Muthukumar *et al*. described the treatment of 3 patients with recurrent tumor progression after surgery (n = 3), and 2 patients who refused surgical treatment or were poor surgical candidates. During the follow-up interval of 1-5 years (median 3 years), 1 patient died of a concurrent illness and all others were stable without deterioration of their clinical condition. One patient exhibited a decrease in lesion volume and 4 patients had no change on follow-up imaging.

We know of no reported radiation-induced toxicity to the brainstem or spinal cord reported in the literature, but radiosurgery does have inherent risks. In 1990, Engenhart *et al*. reported the results of linear accelerator-based radiosurgery in a series of 17 meningiomas.[42] The tumors ranged from 10 to 54 mm in diameter and received single-fraction doses from 10 to 50 Gy. One patient died from brain herniation after radiosurgery (attributed to a shunt malfunction) and another patient died from radiation necrosis and herniation after receiving 35 Gy to a large treatment volume. Five patients developed a large volume of brain edema and brain necrosis was suspected in 3 of them. These complications may be attributed to the use of high dose radiosurgery as the primary modality of treatment of large lesions. We limit our radiosurgical treatment to lesions with a diameter of less than 3.5 cm and use multiple isocenters and three-dimensional image planning.

Limitations of the use of GKRS for foramen magnum meningiomas include difficulties with targeting below the first cervical vertebra. However, this is largely obviated with the current Perfexion version of the Gamma Knife. Additional refinements are necessary to address the cervical spine mobility and target planning in the treatment of lesions below this level. Further limitations of treatment include the necessity of long-term follow-up as the exact rates of recurrence and natural disease progression are not clearly defined.

## CONCLUSIONS

The treatment of skull base meningiomas, whether by open surgical or radiosurgical means, is evolving. Imaging and delivery systems have allowed more accurate targeting and dose planning for radiosurgery. Improved treatment methods and outcomes for these patients is an anticipated result of these advances. Continued outcome reviews and prospective studies are necessary to help develop standardized treatment recommendations for these formidable tumors.

## References

[1] Levy WJ, Bay J, Dohn D (1982). Spinal cord meningioma. J Neurosurg.

[2] George B, Lot G, Boissonnet H (1997). Meningioma of the foramen magnum: A series of 40 cases. Surg Neurol.

[3] Elsberg CA, Strauss I (1929). Tumors of the spinal cord which project into the posterior cranial fossa: Report of a case in which a growth was removed from the ventral and lateral aspects of the medulla oblongata and upper cervical cord. Arch Neurol Psychiatry.

[4] Roberti F, Sekhar LN, Kalavakonda C, Wright DC (2001). Posterior fossa meningiomas: Surgical experience in 161 cases. Surg Neurol.

[5] Adegbite AB, Khan MI, Paine KW, Tan LK (1983). The recurrence of intracranial meningiomas after surgical treatment. J Neurosurg.

[6] Arnautovic KI, Al-Mefty O, Husain M (2000). Ventral foramen magnum meningiomas. J Neurosurg.

[7] Bassiouni H, Ntoukas V, Asgari S, Sandalcioglu EI, Stolke D, Seifert V (2006). Foramen magnum meningiomas: Clinical outcome after microsurgical resection via a posterolateral suboccipital retrocondylar approach. Neurosurgery.

[8] Bruneau M, George B (2008). Foramen magnum meningiomas: Detailed surgical approaches and technical aspects at Lariboisiere Hospital and review of the literature. Neurosurg Rev.

[9] George B, Dematons C, Cophignon J (1988). Lateral approach to the anterior portion of the foramen magnum. Application to surgical removal of 14 benign tumors: Technical note. Surg Neurol.

[10] George B, Lot G, Velut S, Gelbert F, Mourier KL (1993). [French language Society of Neurosurgery. 44th Annual Congress. Brussels, 8-12 June 1993. Tumors of the foramen magnum]. Neurochirurgie.

[11] Goel A, Desai K, Muzumdar D (2001). Surgery on anterior foramen magnum meningiomas using a conventional posterior suboccipital approach: A report on an experience with 17 cases. Neurosurgery.

[12] Kandenwein JA, Richter HP, Antoniadis G (2009). Foramen magnum meningiomas--experience with the posterior suboccipital approach. Br J Neurosurg.

[13] Levy WJ, Latchaw J, Hahn JF, Sawhny B, Bay J, Dohn DF (1986). Spinal neurofibromas: A report of 66 cases and a comparison with meningiomas. Neurosurgery.

[14] Black PM, Villavicencio AT, Rhouddou C, Loeffler JS (2001). Aggressive surgery and focal radiation in the management of meningiomas of the skull base: Preservation of function with maintenance of local control. Acta Neurochir (Wien).

[15] Stafford SL, Perry A, Suman VJ, Meyer FB, Scheithauer BW, Lohse CM (1998). Primarily resected meningiomas: Outcome and prognostic factors in 581 Mayo Clinic patients, 1978 through 1988. Mayo Clin Proc.

[16] Sen CN, Sekhar LN (1990). An extreme lateral approach to intradural lesions of the cervical spine and foramen magnum. Neurosurgery.

[17] Babu RP, Sekhar LN, Wright DC (1994). Extreme lateral transcondylar approach: Technical improvements and lessons learned. J Neurosurg.

[18] Bertalanffy H, Gilsbach JM, Mayfrank L, Klein HM, Kawase T, Seeger W (1996). Microsurgical management of ventral and ventrolateral foramen magnum meningiomas. Acta Neurochir Suppl.

[19] Kratimenos GP, Crockard HA (1993). The far lateral approach for ventrally placed foramen magnum and upper cervical spine tumours. Br J Neurosurg.

[20] George B, Lot G, Boissonnet H (1997). Meningioma of the foramen magnum: A series of 40 cases. Surg Neurol.

[21] Pirotte B, David P, Noterman J, Brotchi J (1998). Lower clivus and foramen magnum anterolateral meningiomas: Surgical strategy. Neurol Res.

[22] Salas E, Sekhar LN, Ziyal IM, Caputy AJ, Wright DC (1999). Variations of the extreme-lateral craniocervical approach: Anatomical study and clinical analysis of 69 patients. J Neurosurg.

[23] Pamir MN, Kilic T, Ozduman K, Ture U (2004). Experience of a single institution treating foramen magnum meningiomas. J Clin Neurosci.

[24] Parlato C, Tessitore E, Schonauer C, Moraci A (2003). Management of benign craniovertebral junction tumors. Acta Neurochir (Wien).

[25] Samii M, Klekamp J, Carvalho G (1996). Surgical results for meningiomas of the craniocervical junction. Neurosurgery.

[26] Nanda A, Vincent DA, Vannemreddy PS, Baskaya MK, Chanda A (2002). Far-lateral approach to intradural lesions of the foramen magnum without resection of the occipital condyle. J Neurosurg.

[27] Meyer FB, Ebersold MJ, Reese DF (1984). Benign tumors of the foramen magnum. J Neurosurg.

[28] Yasuoka S, Okazaki H, Daube JR, MacCarty CS (1978). Foramen magnum tumors. Analysis of 57 cases of benign extramedullary tumors. J Neurosurg.

[29] Guidetti B, Spallone A (1980). Benign extramedullary tumors of the foramen magnum. Surg Neurol.

[30] Zoltan L (1974). [Tumours at the foramen magnum (author's transl)]. Acta Neurochir (Wien).

[31] Love JG, Thelen EP, Dodge HW (1954). Tumors of the foramen magnum. J Int Coll Surg.

[32] Guidetti B, Spallone A (1988). Benign extramedullary tumors of the foramen magnum. Adv Tech Stand Neurosurg.

[33] Stein BM, Leeds NE, Taveras JM, Pool JL (1963). Meningiomas of the foramen magnum. J Neurosurg.

[34] Drummond KJ, Zhu JJ, Black PM (2004). Meningiomas: Updating basic science, management, and outcome. Neurologist.

[35] Kim YJ, Ketter R, Henn W, Zang KD, Steudel WI, Feiden W (2006). Histopathologic indicators of recurrence in meningiomas: Correlation with clinical and genetic parameters. Virchows Arch.

[36] Johnson MD, Sade B, Milano MT, Lee JH, Toms SA (2008). New prospects for management and treatment of inoperable and recurrent skull base meningiomas. J Neurooncol.

[37] Barbaro NM, Gutin PH, Wilson CB, Sheline GE, Boldrey EB, Wara WM (1987). Radiation therapy in the treatment of partially resected meningiomas. Neurosurgery.

[38] Goldsmith BJ, Wara WM, Wilson CB, Larson DA (1994). Postoperative irradiation for subtotally resected meningiomas. A retrospective analysis of 140 patients treated from 1967 to 1990. J Neurosurg.

[39] Wilson CB (1994). Meningiomas: Genetics, malignancy, and the role of radiation in induction and treatment. The Richard C. J Neurosurg.

[40] Nicolato A, Foroni R, Pellegrino M, Ferraresi P, Alessandrini F, Gerosa M (2001). Gamma knife radiosurgery in meningiomas of the posterior fossa. Experience with 62 treated lesions. Minim Invasive Neurosurg.

[41] Muthukumar N, Kondziolka D, Lunsford LD, Flickinger JC (1999). Stereotactic radiosurgery for anterior foramen magnum meningiomas. Surg Neurol.

[42] Engenhart R, Kimmig BN, Hover KH, Wowra B, Sturm V, van Kaick G (1990). Stereotactic single high dose radiation therapy of benign intracranial meningiomas. Int J Radiat Oncol Biol Phys.

